# Radiologists’ diagnostic confidence in surgically referred pulmonary nodules: a routine computed tomography report retrospective analysis

**DOI:** 10.1186/s12890-026-04336-6

**Published:** 2026-05-18

**Authors:** Marina Higashi, Rintaro Ito, Shingo Iwano, Shinichiro Kamiya, Taketo Kato, Shota Nakamura, Tetsuya Mizuno, Toyofumi Fengshi Chen-Yoshikawa, Shinji Naganawa

**Affiliations:** 1 Department of Radiology, Nagoya University Graduate School of Medicine, 65 Tsurumai-Cho, Showa-Ku, Nagoya, 466-8550 Japan; 2 Department of Innovative BioMedical Visualization, Nagoya University Graduate School of Medicine, 65 Tsurumai-Cho, Showa-Ku, Nagoya, 466-8550 Japan; 3 Medical Imaging Engineering, Department of Integrated Health Sciences, Nagoya University Graduate School of Medicine, 1-1-20 Daiko-Minami, Higashi-Ku, Nagoya, 461-8673 Japan; 4 Department of Thoracic Surgery, Nagoya University Graduate School of Medicine, 65 Tsurumai-Cho, Showa-Ku, Nagoya, 466-8550 Japan

**Keywords:** Lung, Pulmonary nodules, Tomography, Computed, Diagnostic accuracy, Radiology reports

## Abstract

**Background:**

Imaging plays a critical role in the preoperative evaluation of pulmonary nodules, especially when obtaining a definitive biopsy is not feasible. This study evaluated the reliability of routine computed tomography (CT) reports in differentiating benign from malignant nodules and to identify imaging features that influenced radiologists' diagnostic confidence.

**Methods:**

We retrospectively analyzed 823 patients who underwent preoperative CT for pulmonary nodules at a single institution between 2017 and 2023. Radiologists' diagnostic confidence levels, recorded on a five-point scale (1 = benign, 5 = malignant), were extracted from reports and compared with pathological diagnoses. Diagnostic performance was assessed using the area under the curve (AUC) and risk of malignancy (ROM).

**Results:**

A total of 823 pulmonary lesions were evaluated, including 701 malignant and 122 benign nodules. Radiologists' interpretations demonstrated excellent diagnostic performance, with an AUC of 0.961; 95%CI: 0.94—0.98, sensitivity of 0.95, and specificity of 0.86. Notably, 99.4% of nodules rated "5" were confirmed malignant. Diagnostic accuracy was significantly higher for ground-glass nodules compared with solid nodules (AUC 0.995 vs. 0.938, *p* < 0.001) and when prior CT scans were available (AUC 0.983 vs. 0.951, *p* = 0.036). The presence of coexisting chronic obstructive pulmonary disease or interstitial lung disease modestly reduced accuracy (AUC 0.973 vs. 0.919, *p* = 0.089).

**Conclusion:**

Radiologists’ diagnostic confidence levels recorded in routine preoperative CT reports demonstrated high concordance with pathological outcomes in pulmonary nodules referred for diagnostic surgical resection after nondiagnostic biopsy, suggesting that clinical decision-making in this highly selected population is generally reliable.

## Introduction

Lung cancer is the leading cause of cancer-related mortality worldwide [1]. For patients with resectable lung cancer, surgical resection is the standard treatment modality [2] In routine practice, a definitive histopathological diagnosis is typically established preoperatively using bronchoscopy or CT-guided biopsy. Despite these procedures, a substantial proportion of pulmonary nodules remain diagnostically indeterminate. This is often because certain nodules are deemed unsuitable for biopsy due to technical complexities or prohibitive procedural risks. In such situations, patients are often referred for diagnostic surgical resection based on a comprehensive clinical assessment, including CT findings, prior imaging follow-up, PET/CT results, and multidisciplinary discussion [3, 4].

Previous studies have mainly focused on the diagnostic performance of low-dose CT in lung cancer screening or on radiomics and deep learning approaches applied to curated imaging datasets [5–10]. In contrast, little is known about how accurately radiologists’ routine interpretations support clinical decision-making in diagnostically challenging nodules that are ultimately referred for surgery [11].

At high-volume, tertiary referral centers specializing in lung cancer care, diagnostic resection is considered for pulmonary nodules that remain indeterminate after nondiagnostic biopsy or are technically infeasible to biopsy. For these strictly selected cases, the radiology reports generated during the preoperative evaluation process reflect the radiologist’s comprehensive assessment.

Accordingly, this study aimed to evaluate the reliability of radiologists’ diagnostic confidence recorded in routine preoperative CT reports for pulmonary nodules referred for diagnostic surgical resection after nondiagnostic or infeasible biopsy. A secondary objective was to identify specific imaging features described in the CT reports that most strongly influenced radiologists’ confidence in classifying lesions as benign or malignant.

## Materials and methods

This retrospective study was approved by our Institutional Review Board, with a waiver of informed consent for retrospective data analysis (Approval No.2024—0304).

### Study population

This retrospective study was conducted at a single tertiary referral center. Using the search function of the Picture Archiving and Communication System (PACS), we identified patients who underwent dynamic contrast-enhanced CT ordered by thoracic surgeons for preoperative evaluation of pulmonary lesions between January 2017 and December 2023. We included patients aged ≥ 20 years who underwent preoperative CT for pulmonary masses or nodules. Initial case identification was based on a review of radiology reports and subsequently confirmed by reviewing corresponding electronic medical records. All malignant diagnoses were pathologically confirmed via surgery or biopsy. Cases with definitive histopathological or clinical diagnoses of benign or malignant tumors were included. Patients were excluded based on the following criteria: 1) diagnosis of malignancy confirmed by biopsy prior to the CT scan; 2) presence of lung metastases, recurrent lung cancer, or lack of a definitive pathological or clinical diagnosis; and 3) radiology reports that did not contain an assessment of benignity versus malignancy.

**Fig. 1:**
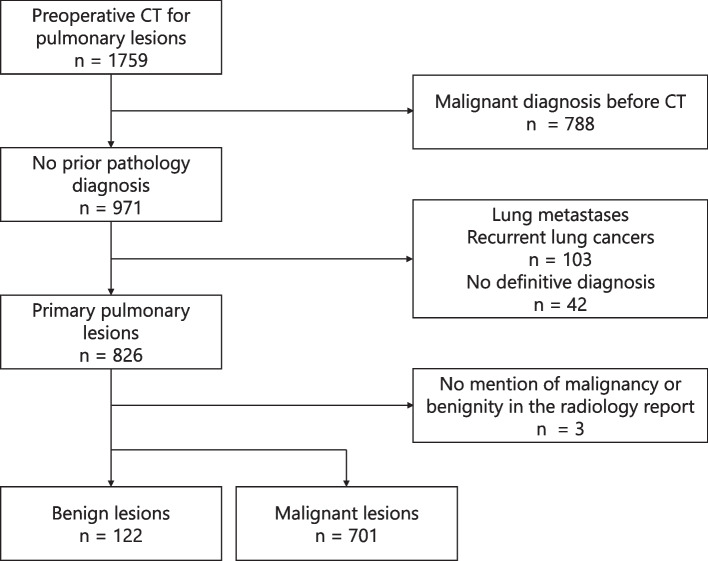
A flowchart of the patient selection process

After applying these exclusion criteria, 823 patients were included in the final analysis. This cohort consisted of 468 men and 355 women, with ages ranging from 22 to 91 years (mean age: 67.7 ± 11.0 years). A flowchart of the patient selection process is shown in Fig. 1.

### CT scan and reporting

CT images were acquired using two scanners during the study period. A dual-source CT scanner (SOMATOM Definition Flash; Siemens Healthcare, Tokyo, Japan) was used between 2017 and 2019, and a high-definition CT scanner (Aquilion Precision; Canon Medical Systems Corp., Otawara, Japan) was used between 2020 and 2023.

All scans were performed as dual-phase dynamic contrast-enhanced chest CTs, including both arterial and venous phases, following intravenous administration of iodinated contrast material. At our institution, all patients with suspected pulmonary mass lesions routinely undergo dual-phase dynamic contrast-enhanced chest CT as part of preoperative evaluation. Images were reconstructed using two different settings. For lung parenchyma evaluation, images were reconstructed at a slice thickness of 0.5–0.6 mm with a high-frequency (sharp) reconstruction kernel. For mediastinal and soft tissue evaluation, images were reconstructed at a 1.0—mm slice thickness with a standard (smooth) algorithm.

All CT images were interpreted as part of routine clinical practice by one or two board-certified diagnostic radiologists. At our institution, it is standard practice to include an assessment of the likelihood of pulmonary nodules or mass malignancy in radiology reports using a five-point confidence scale [11, 12]. Confidence levels were defined as follows:

- 1 = benign;
- 2 = Probably benign;
- 3 = Indeterminate;
- 4 = Probably malignant;
- 5 = Malignant.

Radiology reports were retrospectively reviewed, and the assigned confidence levels were extracted for each case. These confidence scores were then used to calculate the risk of malignancy (ROM) and to evaluate diagnostic performance through statistical analysis, including receiver operating characteristic (ROC) curve analysis.

In addition, the following clinical and imaging data were extracted from radiology reports and corresponding medical records: age, sex, maximum tumor diameter, diameter of the solid component (if present), and lesion type, classified as either ground-glass nodules (GGN) or solid nodules (SN). The presence or absence of spiculation and pleural indentation was also recorded, along with documentation of coexisting chronic obstructive pulmonary disease (COPD) and/or interstitial lung disease (ILD). Furthermore, data from the Picture Archiving and Communication System (PACS) were reviewed to determine whether CT images obtained more than three months before the diagnostic CT were available. Information on whether PET/CT had been performed prior to the diagnostic CT, either at our institution or at an outside facility, was also collected.

### Statistical analysis

All statistical analyses were performed using IBM SPSS Statistics, version 30 (IBM Corp., Armonk, NY, USA) and R version 4.5.1 (The R Foundation for Statistical Computing). A two-sided *p*-value of < 0.05 was considered statistically significant.

The ROM was calculated for each of the five confidence levels (1 = benign to 5 = malignant) to evaluate the concordance between radiologists’ diagnostic certainty and final pathological or clinical diagnoses. The consolidation-to-tumor (C/T) ratio was calculated as the ratio of the maximum diameter of the solid portion to the maximum overall tumor diameter, based on radiology reports. ROC curve analysis was used to evaluate the accuracy of radiologists’ confidence levels in differentiating between benign and malignant pulmonary lesions, and the area under the curve (AUC) was calculated. The optimal cutoff value for dichotomizing the five-point confidence levels was determined using the Youden Index, and sensitivity (SEN) and specificity (SPE) were calculated. We first performed ROC analysis for all cases. Subsequently, subgroup analyses were conducted to compare diagnostic performance across various clinical and imaging factors. Differences in the AUCs between subgroups were tested using the non-parametric DeLong test. Logistic regression analysis was performed to identify imaging features documented in the radiology reports that were significantly associated with lesion malignancy. This analysis was conducted for both the entire cohort and the solid-nodule subgroup.

## Results

Of the 823 pulmonary lesions included in the final analysis, 701 (85.2%) were malignant and 122 (14.8%) were benign. All malignant lesions were histopathologically confirmed and included 583 adenocarcinomas, 75 squamous cell carcinomas, and 43 other histological subtypes of lung cancer. Among the benign lesions, 86 were diagnosed histopathologically, whereas the remaining 36 were diagnosed clinically based on follow-up imaging that demonstrated either lesion size reduction or stability for at least two years. Details are presented in Table 1. The size of the pulmonary lesions ranged from 5 to 115 mm, with a mean diameter of 24.9 ± 14.3 mm. Of the 823 lesions, 372 (45.2%) were classified as GGNs and 426 (51.8%) as SNs. In 25 patients (3.0%), the nodule type (GGN or SN) was not documented in the radiology report.

**Table 1:** Final diagnosis of pulmonary nodules

	**Final Diagnosis**	**n**
Malignant	Adenocarcinoma	583
	Squamous cell carcinoma	75
	Large cell carcinoma	10
	Small cell carcinoma	8
	Pleomorphic carcinoma	5
	Carcinoid	4
	Adenosquamous carcinoma	3
	Non-small cell carcinoma	3
	Large cell neuroendocrine carcinoma	3
	Sarcoma	2
	Lymphoma	2
	Lymphoepithelioma-like carcinoma	1
	Sarcomatoid carcinoma	1
	Methotehelioma	1
Benign	Granuloma	12
	Inflammation	9
	Pulmonary sequestration	9
	Non-tuberculous mycobacterial disease	8
	Aspergillosis	7
	Hamartoma	5
	Pulmonary arteriovenous fistula	5
	Fibrosis	3
	Sclerosing pneumocytoma	3
	Solitary fibrous tumor	3
	Cryptococcosis	3
	Organizing pneumonia	2
	Pneumonia	2
	Congenital pulmonary airway malformation	2
	Intrapulmonary lymph node	2
	Hemangioma	1
	Hematoma	1
	Inflammatory myofibroblastic tumor	1
	Nodular lymphoid hyperplasia	1
	Abscess	1
	Mycosis	1
	Myelolipoma	1
	Bronchial atresia	1
	No malignancy	1
	Shrinkage or disappearance after follow-up	21
	Solid nodule that remained unchanged for over 2 years	15

The distribution of cases according to radiologists’ confidence levels is summarized in Table 2. A total of 48 patients (5.8%) were classified as level 1, 48 (5.8%) as level 2, 44 (5.3%) as level 3, 148 (18.0%) as level 4, and 535 (65.0%) as level 5. The corresponding ROM for each category was 4.2% for level 1, 12.5% for level 2, 61.4% for level 3, 90.5% for level 4, and 99.4% for level 5. Regarding diagnostic discrepancies, one case of sarcoma and one case of combined pulmonary aspergillosis and large cell carcinoma were misdiagnosed as benign at confidence level 1. At level 2, false-negative findings included three cases of adenocarcinoma, and one case each of squamous cell carcinoma, sarcoma, and a combination of large cell neuroendocrine carcinoma and granuloma. Conversely, at level 5, three cases were misdiagnosed as malignant (false-positive), which histologically consisted of two granulomas and one pulmonary mycosis.

**Table 2:** The distribution of cases according to radiologists’ diagnostic confidence levels

Confidence Level	1 benign	2 Probably benign	3 Indeterminate	4 Probably malignancy	5 Malignant
N	48	48	44	148	535
Benign/Malignant (N)	46/2	42/6	17/27	14/134	3/532
ROM (%)	4.2	12.5	61.4	90.5	99.4
Female/Male (N)	23/25	16/32	22/22	63/85	231/304
Age (years)	53.0 ± 14.0	62.9 ± 13.5	67.0 ± 10.7	67.9 ± 10.1	69.4 ± 9.5
Total size (mm)	40.3 ± 28.5	22.8 ± 16.5	17.5 ± 15.1	19.7 ± 10.1	26.3 ± 12.9
GGN/SN/Not described (N)	0/29/19 (0%/60%/40%)	3/42/3 (6%/88%/6%)	7/37/0 (16%/84%/0%)	77/79/2 (16%/84%/0%)	295/239/1 (55%/45%/0%)
C/T ratio (%)	100.0 ± 0.0	94.7 ± 21.5	90.6 ± 23.4	70.8 ± 37.6	69.1 ± 34.1
Spicula (N)	1 (2%)	7 (15%)	8 (18%)	34 (23%)	125 (23%)
Pleural Indent (N)	1 (2%)	8 (17%)	11 (25%)	60 (41%)	236 (44%)
COPD (N)	3 (6%)	13 (27%)	12 (27%)	38 (26%)	168 (31%)
ILD (N)	0 (0%)	3 (6%)	2 (5%)	13 (9%)	40 (7%)
Past CT (N)	11 (23%)	20 (42%)	18 (41%)	58 (39%)	191 (36%)
PET/CT (N)	12 (25%)	31 (65%)	31 (70%)	111 (75%)	419 (78%)

*C/T ratio* Consolidation to tumor ratio, *COPD* Chronic obstructive pulmonary disease, *GGN* Ground-glass nodule, *ILD* Interstitial lung disease, *SN* Solid nodule, *ROM* Risk of malignancy

**Fig. 2:**
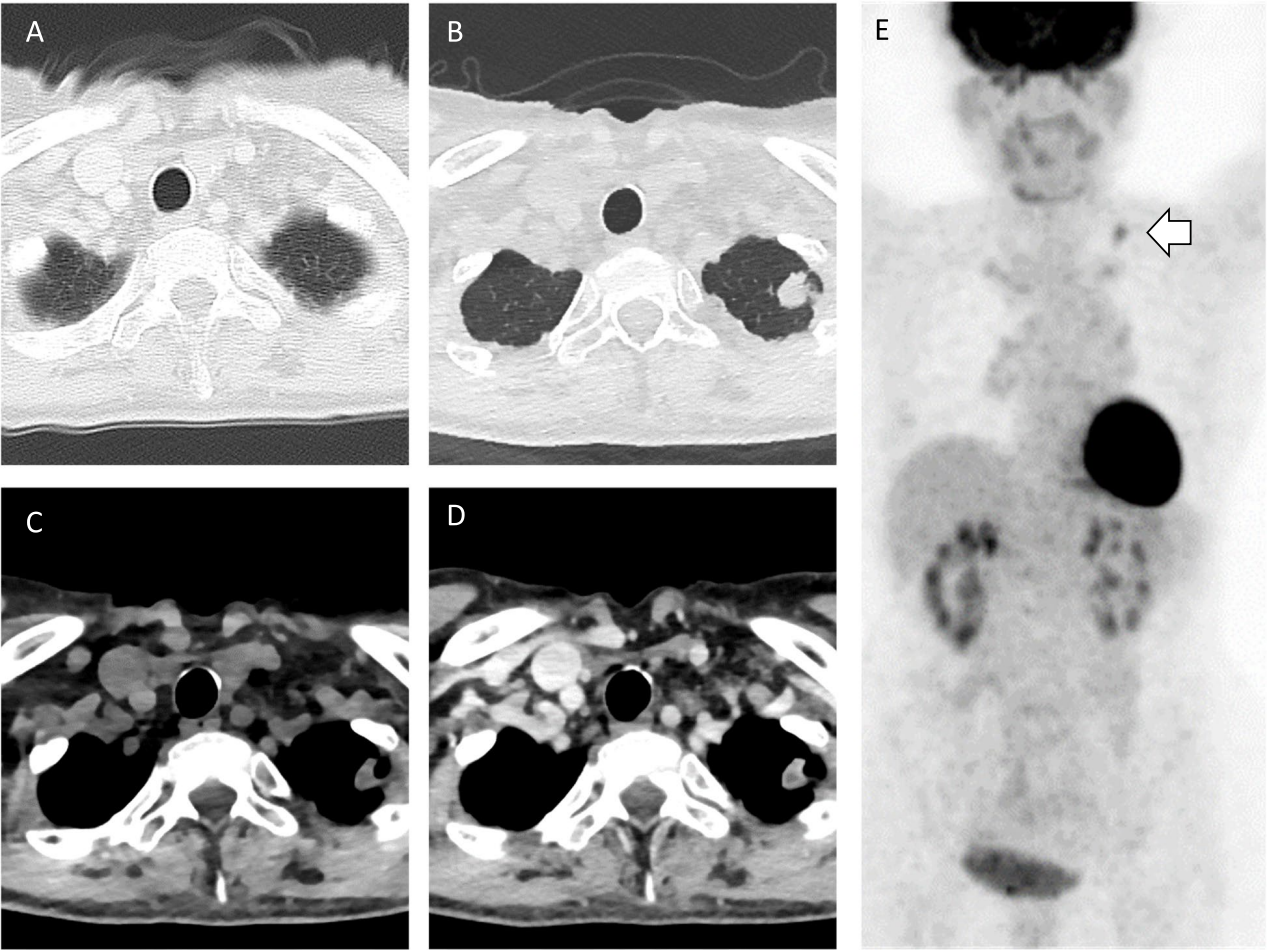
A case of confidence level 1 (Benign). A 57-year-old man. 1.5 cm solid nodule in the apex of the left upper lobe. Contrast-enhanced CT showed a non-enhancing necrotic area. PET revealed FDG uptake slightly higher than that of the liver. The lesion was absent in a CT scan taken three months prior, leading to a diagnosis of inflammation. Pathological examination of the surgical specimen confirmed the diagnosis of Epithelioid granuloma. **A** Axial non-contrast CT with lung window settings, 3 months ago. **B** Axial non-contrast CT with lung window settings. **C** Axial non-contrast CT with mediastinal window settings. **D** Axial contrast CT with mediastinal window settings. **E** Coronal 18F-FDG PET/CT scan, a nodule is present at the location of the arrow

**Fig. 3:**
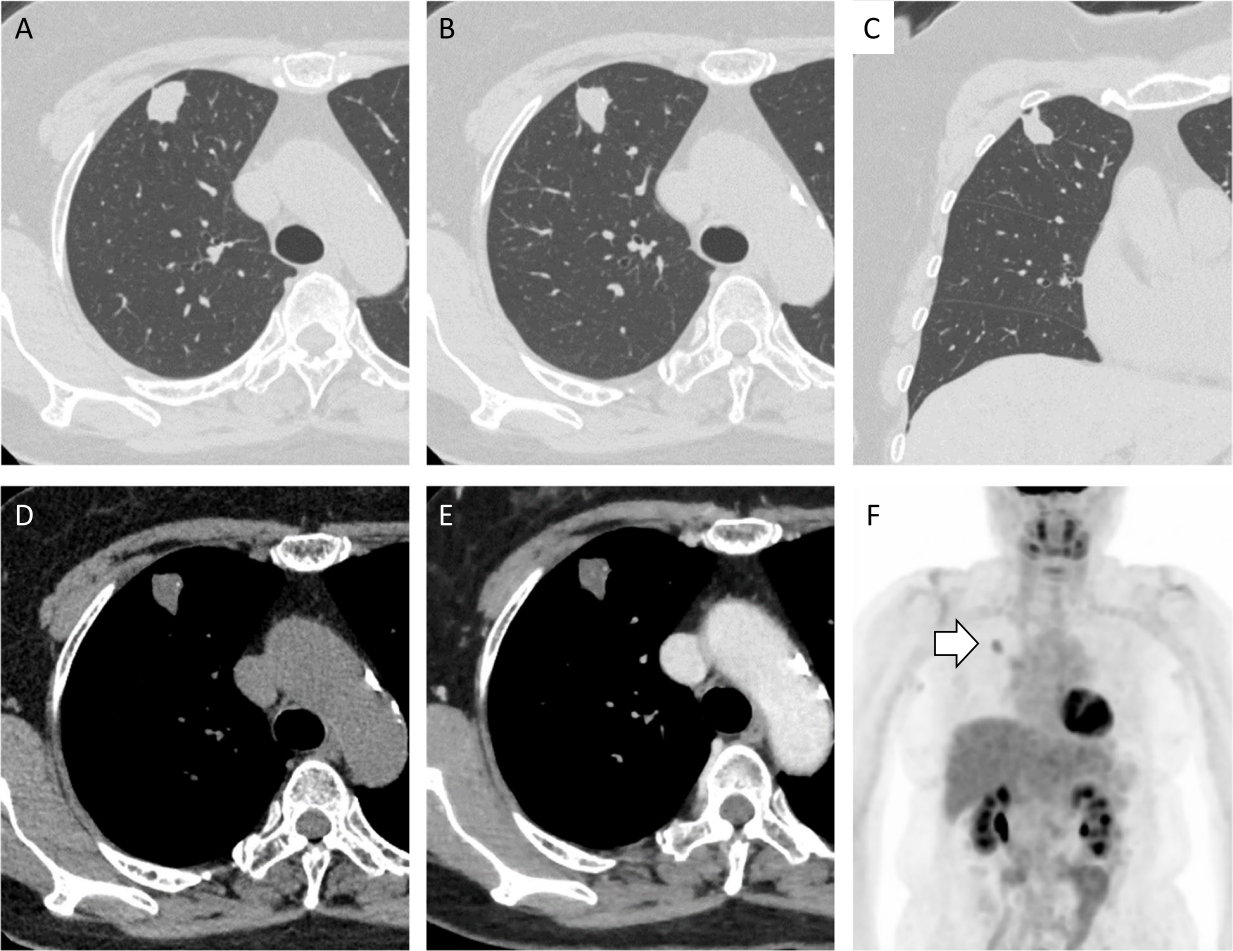
A case of confidence level 2 (Probably benign). A 70-year-old woman. A 2.0 cm solid nodule in the right upper lobe. Part of the margin appears linear. Pleural indentation and calcification are observed. The lesion shows poor contrast enhancement, suggesting that a large portion consists of necrotic or mucinous components. A previous PET scan at the referring institution showed FDG uptake slightly lower than that of the liver. Based on its shape and enhancement pattern, non-tuberculous mycobacterial (NTM) infection was suspected. Pathological examination of the surgical specimen confirmed the diagnosis of NTM. **A** Axial non-contrast CT with lung window settings. Pleural indentation is observed. **B** Axial non-contrast CT with lung window settings. Calcification is observed. **C** Coronal non-contrast CT with lung window settings. **D** Axial non-contrast CT with mediastinal window settings. **E** Axial contrast CT with mediastinal window settings. **F** Coronal 18F-FDG PET/CT scan, a nodule is present at the location of the arrow

**Fig. 4:**
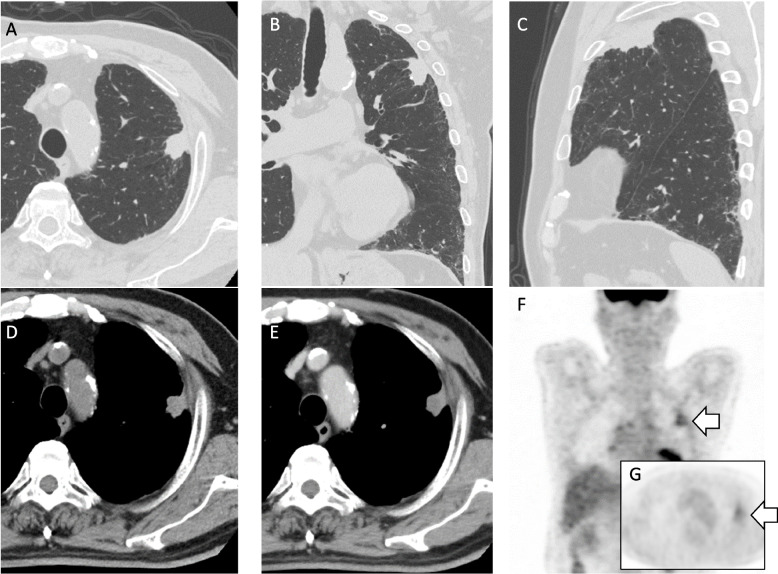
A case of confidence level 3 (Indeterminate). A 72-year-old man. A 5.6 cm solid mass located just beneath the chest wall in the left upper lobe. The mass is lobulated with spiculation. Mild contrast enhancement is observed at the peripheral region of the mass. A previous PET at the referring institution showed FDG uptake comparable to that of the liver. Nontuberculous mycobacterial infection and primary lung cancer were considered as differential diagnoses. Pathological examination of the surgical specimen confirmed the diagnosis of nontuberculous mycobacterial infection. **A** Axial non-contrast CT with lung window settings. **B** Coronal non-contrast CT with lung window settings.** C** Sagittal non-contrast CT with lung window settings. **D** Axial non-contrast CT with mediastinal window settings. **E** Axial contrast CT with mediastinal window settings. **F** Coronal 18F-FDG PET/CT scan, a nodule is present at the location of the arrow. **G** Axial 18F-FDG PET/CT scan, a nodule is present at the location of the arrow

**Fig. 5:**
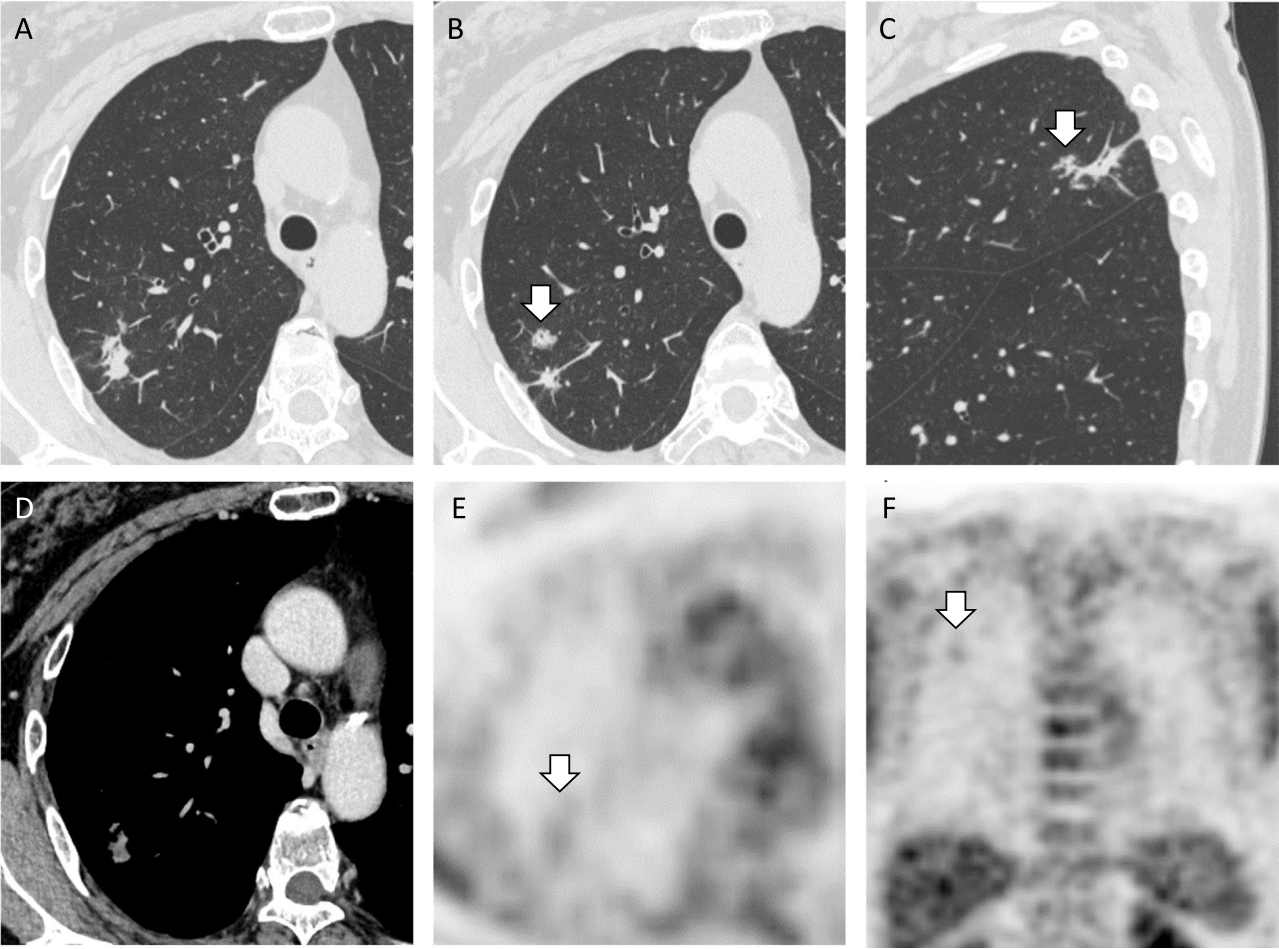
A case of confidence level 4 (Probably malignant). A 63-year-old woman. A 2.8 cm solid nodule in the right upper lobe. A faint ground-glass opacity was seen anterior to the nodule (arrows of B, C). PET/CT from the previous hospital showed mild FDG uptake (arrows of E, F). While lung adenocarcinoma was suspected, the irregular morphology on sagittal images suggested an inflammatory scar. Pathological examination of the surgical specimen confirmed the diagnosis of Adenocarcinoma, pT1cN0. **A** Axial non-contrast CT with lung window settings. **B** Axial non-contrast CT with lung window settings, which represents a more anterior slice than A. **C** Sagittal non-contrast CT with lung window settings. **D** Axial contrast CT with mediastinal window settings. **E** Axial 18F-FDG PET/CT scan. **F** Coronal 18F-FDG PET/CT scan

**Fig. 6:**
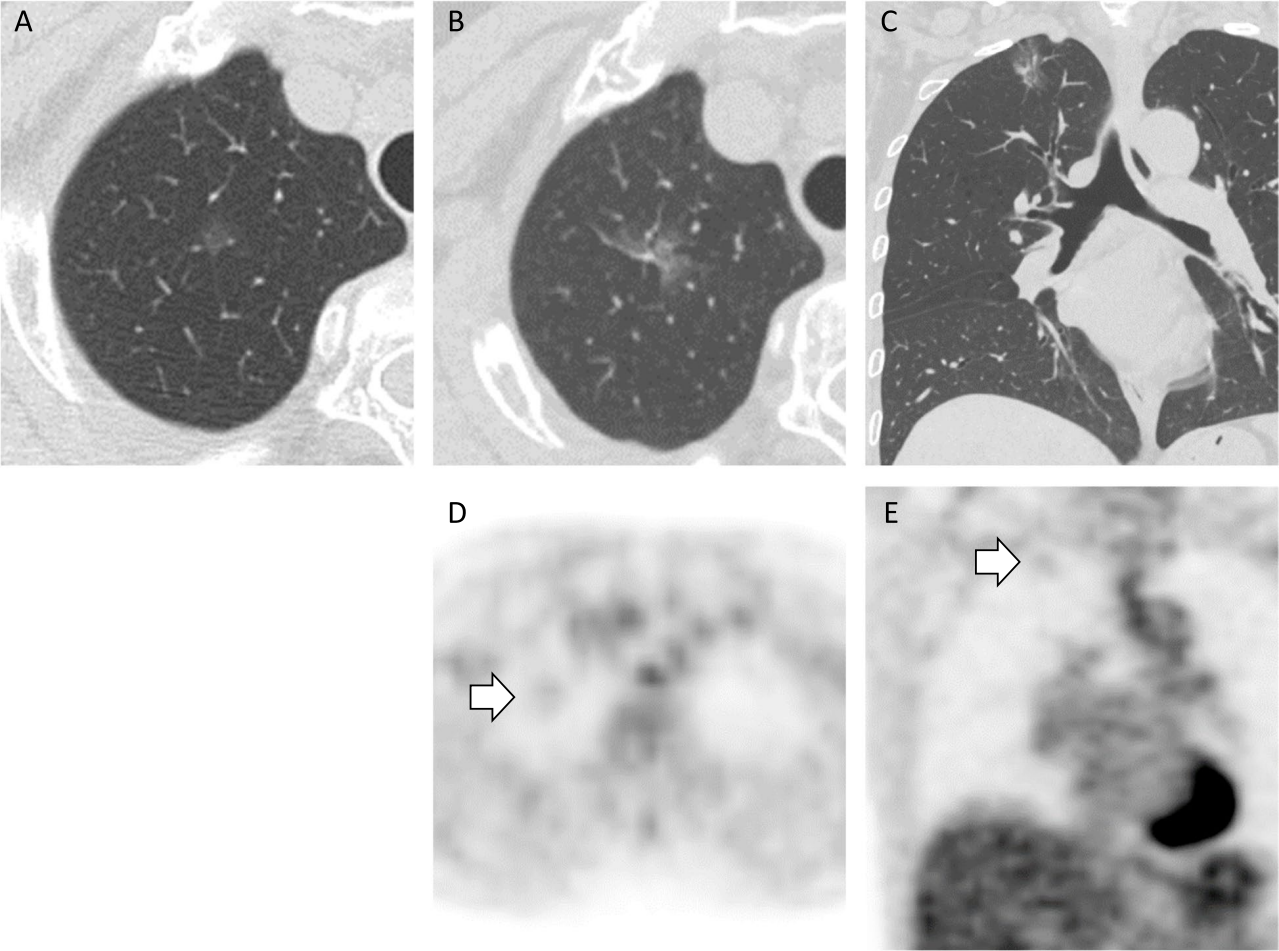
A case of confidence level 5 (Malignant). A 76-year-old woman. A 2.0 cm part-solid GGN in the right upper lobe. PET/CT showed mild FDG uptake (arrows of E, F). A pure GGN had been identified on CT eight years earlier, which had since enlarged, leading to a diagnosis of primary lung cancer. Pathological examination of the surgical specimen confirmed the diagnosis of Adenocarcinoma, pT1aN0. **A** Axial non-contrast CT with lung window settings, 8 years ago. **B** Axial non-contrast CT with lung window settings. **C** Coronal non-contrast CT with lung window settings. **D** Axial 18F-FDG PET/CT scan. **E** Coronal 18F-FDG PET/CT scan

**Fig. 7:**
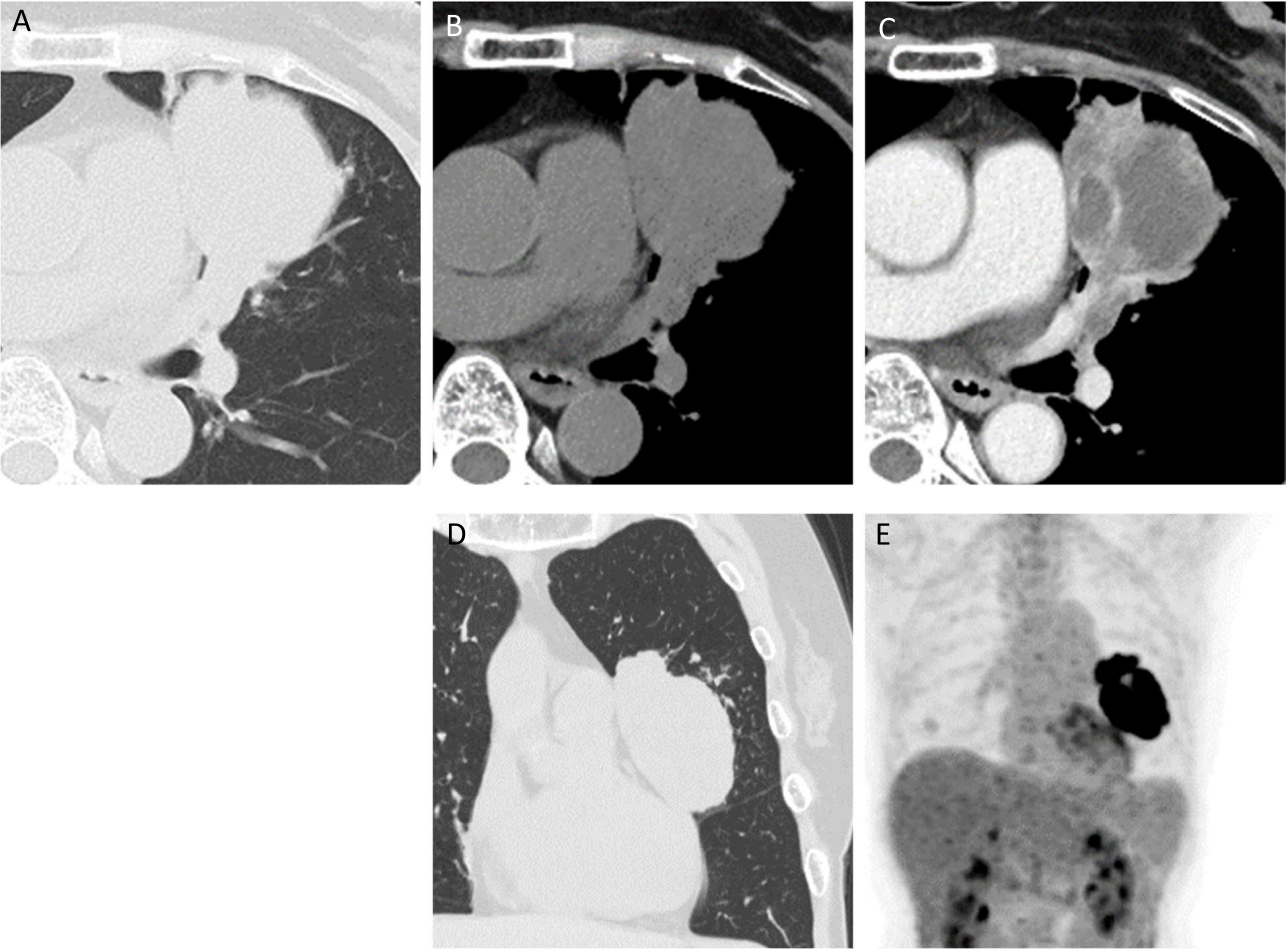
A misdiagnosed case of confidence level 1 (benign). A 60-year-old woman. A 6.9 cm solid mass in the left upper lobe. The patient was referred for surgery after failing to respond to antimicrobial treatment for a smear-positive mycobacterial infection at the previous hospital. Therefore, we initially diagnosed the lesion as aspergillosis. However, PET/CT performed after CT revealed intense FDG uptake. Additionally, cytology from the referring hospital reported class V findings, leading to a diagnosis of primary lung cancer, which was surgically resected. Pathological examination of the surgical specimen confirmed the diagnosis of pulmonary aspergillosis and large cell carcinoma. **A** Axial non-contrast CT with lung window settings. **B** Axial non-contrast CT with mediastinal window settings. **C** Axial contrast CT with mediastinal window settings. **D** Coronal contrast CT with mediastinal window settings. **E** Coronal 18F-FDG PET/CT scan

**Fig. 8:**
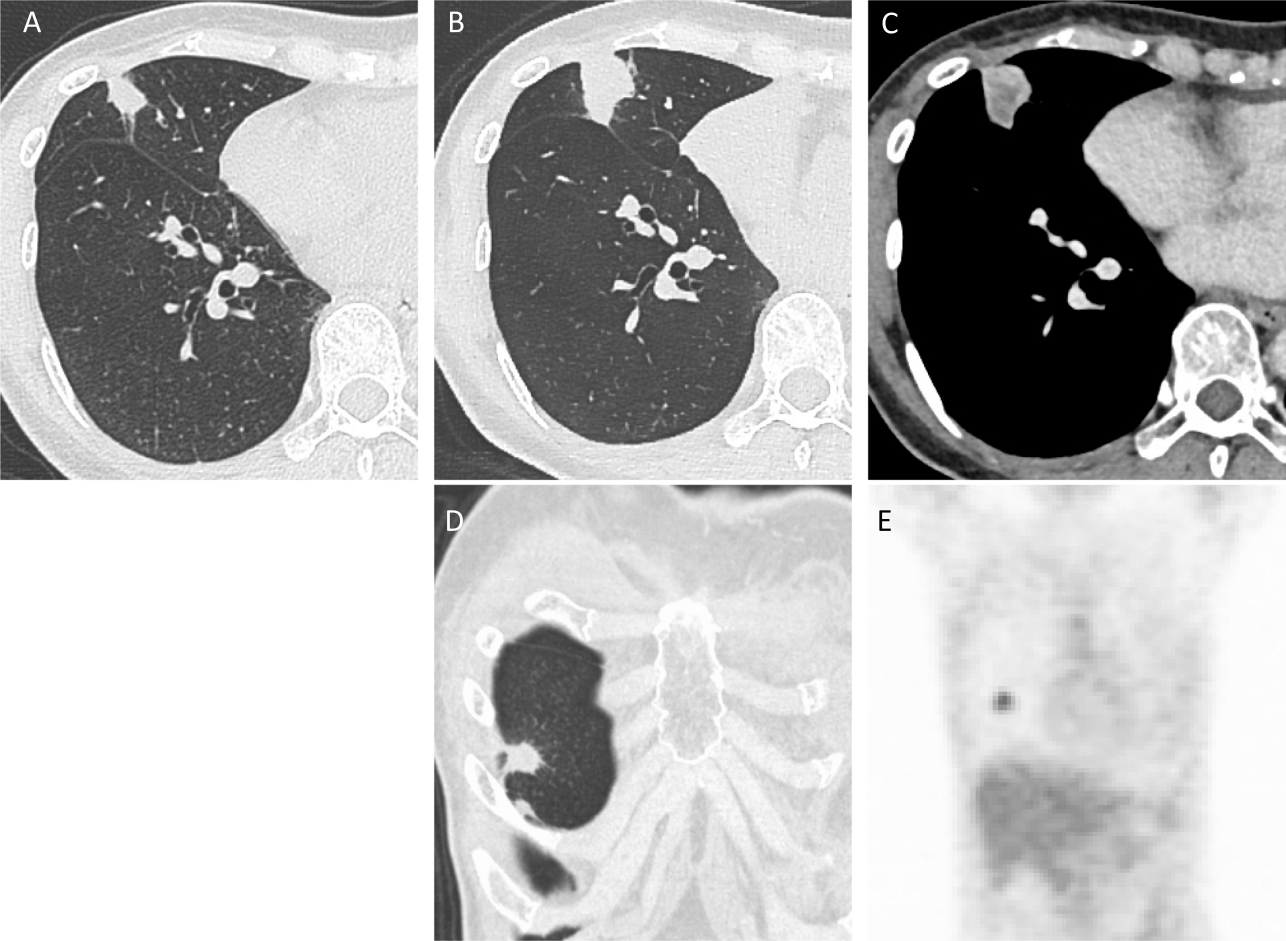
A misdiagnosed case of confidence level 5 (malignant). A 54-year-old woman. A 2.4 cm solid nodule in the right middle lobe, accompanied by spiculation and pleural indentation. A non-enhancing area is observed in the central region. Compared to the previous CT from the referring institution eight months ago, the nodule has increased in size. PET/CT showed FDG uptake higher than that of the liver, suggesting primary lung cancer. Pathological examination of the surgical specimen confirmed the diagnosis of granuloma. **A** Axial non-contrast CT with lung window settings, 8 months ago. **B** Axial non-contrast CT with lung window settings. **C** Axial contrast CT with mediastinal window settings. **D** Coronal contrast CT with mediastinal window settings. **E** Coronal 18F-FDG PET/CT scan

Figures 2, 3, 4, 5 and 6 presents cases for each confidence level. Misdiagnosed cases are presented in Figs. 7 and 8.

**Fig. 9:**
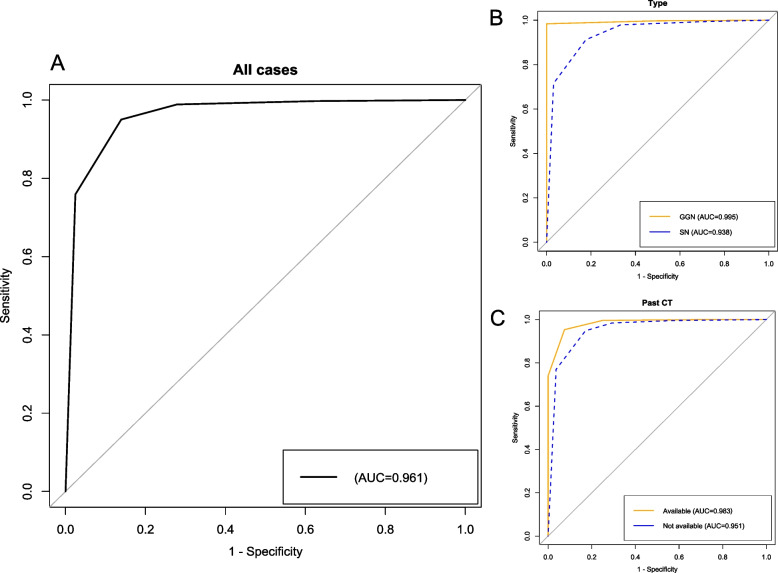
ROC curves. ROC curves showing the diagnostic value in (**A**) all cases, (**B**) ground-glass nodules and solid nodules, and (**C**) cases with and without prior CT scans

The ROC analysis demonstrated excellent diagnostic performance for differentiating between benign and malignant lesions (Table 3 and Fig. 9). The AUC for all cases was 0.961 (95% confidence interval [CI]: 0.942–0.980). Based on the Youden Index, the optimal cutoff value was a confidence level of 4; levels 1–3 were classified as benign, and levels 4–5 as malignant. Using this threshold, the sensitivity and specificity were found to be 0.950 and 0.861, respectively.

**Table 3:** Area under the curve of receiver operating characteristic analysis for differentiation between benign and malignant pulmonary lesions

	**N**	**AUC**	**95% CI**		***p* value**	**SEN**	**SPE**
All	823	0.961	0.942	0.980		0.950	0.861
Period							
2017—2019	288(35%)	0.964	0.929	0.999	0.816	0.956	0.865
2020—2023	535(65%)	0.959	0.936	0.982		0.947	0.859
Age							
≦60 years	190(23%)	0.946	0.913	0.980	0.502	0.909	0.899
> 60 years	633(77%)	0.961	0.935	0.987		0.959	0.811
Size							
≦1.0 cm	56(7%)	0.882	0.795	0.970	0.120*	0.718	0.882
1.1—2.0 cm	312(38%)	0.951	0.909	0.994	0.823*	0.950	0.848
> 2.0 cm	431(52%)	0.957	0.925	0.990		0.850	0.960
Not described	24(3%)						
Type							
GGN	372(45%)	0.995	0.988	1.000	< 0.001	0.984	1.000
SN	426(52%)	0.938	0.911	0.965		0.912	0.823
Not described	25(3%)						
Spicula							
With	174(21%)	0.878	0.778	0.977	0.065	0.778	0.875
Without	649(79%)	0.973	0.959	0.988		0.948	0.906
Pleural Indent							
With	316(38%)	0.953	0.926	0.981	0.716	0.795	1.000
Without	507(62%)	0.960	0.937	0.983		0.933	0.903
COPD/ILD							
With	254(31%)	0.919	0.858	0.979	0.089	0.779	0.929
Without	569(69%)	0.973	0.957	0.989		0.949	0.915
Past CT							
Available	298(36%)	0.983	0.970	0.997	0.036	0.953	0.925
Not available	525(64%)	0.951	0.924	0.978		0.948	0.829
PET/CT							
Available	604(73%)	0.942	0.908	0.975	0.058	0.956	0.781
Not available	219(27%)	0.978	0.961	0.994		0.932	0.948

^*^against > 2.0 cm

*AUC* Area under the curve, *COPD* Chronic obstructive pulmonary disease, *GGN* Ground-glass nodule, *ILD* Interstitial lung disease, *SN* Solid nodule, *SEN* Sensitivity, *SPE* Specificity, *CI* Confidential interval

Subgroup analysis of the ROC curves revealed that GGNs had significantly higher diagnostic performance than solid nodules, with AUCs of 0.995 and 0.938, respectively (*p* < 0.001; Table 3). In addition, cases with prior CT images available showed significantly higher diagnostic accuracy compared to those without prior imaging (AUC 0.983 vs. 0.951, *p* = 0.036, Table 3). Patients without coexisting COPD or ILD showed a trend toward higher diagnostic performance than those with these comorbidities (AUC 0.973 vs. 0.919, *p* = 0.089, Table 3). Of the cases with available PET/CT data, 291 PET/CT scans were performed at our institution, while 310 were performed at other hospitals. A sub-analysis was conducted on 426 cases with solid nodules to evaluate the diagnostic performance based on the presence or absence of PET/CT data. In the PET/CT group (*n* = 328), the AUC was 0.925 (95% CI: 0.886–0.965), with a sensitivity of 0.738 and a specificity of 0.947. In the non-PET group (*n* = 98), the AUC was 0.937 (95% CI: 0.890–0.983), with a sensitivity of 0.831 and a specificity of 0.923. There was no significant difference between the two AUCs (*p* = 0.718).

Logistic regression analysis demonstrated that the radiologists’ confidence levels, as recorded in the CT reports, were significant predictors of lesion malignancy in both the overall cohort and the solid nodule subgroup (Table 4). The odds ratio (OR) for confidence level was 8.509 for all lesions and 6.132 for solid nodules (both *p* < 0.05). Other significant predictors of malignancy included patient age, C/T ratio, presence of spiculation, pleural indentation, and coexisting COPD and/or ILD (Table 4).

**Table 4:** Factors correlating with diagnosis of benign and malignant

	**ALL**		**SN**	
	Odds Ratio	*P* value	Odds Ratio	*P* value
Confidence Level	8.509	< 0.001	6.132	< 0.001
Age	1.074	< 0.001	1.074	< 0.001
Size (mm)	0.993	0.290	0.988	0.742
C/T ratio (%)	0.948	< 0.001	N/A	N/A
Spicula	1.928	0.020	3.684	0.001
Pleural Indent	3.985	< 0.001	2.568	< 0.001
COPD and/or ILD	4.068	0.044	3.477	< 0.001

*C/T ratio* Consolidation to tumor ratio, *COPD* Chronic obstructive pulmonary disease, *ILD* Interstitial lung disease, *SN* Solid nodule

## Discussion

In this study, we demonstrated that radiology reports provided by diagnostic radiologists have a high diagnostic accuracy for pulmonary nodules, with an overall AUC of 0.96, sensitivity of 0.95, and specificity of 0.86. The diagnostic performance was particularly strong for ground-glass nodules, and was enhanced when prior CT images were available, but modestly reduced in patients with diffuse lung disease.

This study had several notable strengths. First, our analysis was based on real-world data from routine clinical radiology reports, which enhances the external validity and generalizability of the findings. Second, the study included a large cohort of 823 cases collected over a seven-year period, providing robust statistical power. Third, the final diagnoses were highly reliable, as most cases were pathologically confirmed. Finally, the implementation of a five-point diagnostic confidence scale in radiology reports enabled a detailed, quantitative evaluation of diagnostic certainty.

Previous studies have developed various predictive models to differentiate between benign and malignant pulmonary lesions, and several meta-analyses have evaluated their diagnostic performance. For example, a meta-analysis of 27 studies using CT-based radiomics approaches yielded a pooled AUC of 0.91 [10]. Similarly, a review of eight studies focusing on PET/CT radiomics reported a pooled AUC of 0.89 [8]. Furthermore, a meta-analysis by Mao et al., evaluating 27 studies on Lung-RADS version 1.0, reported a pooled sensitivity of 0.96 and a specificity of 0.90 for diagnosing lung cancer [13]. For comparison, another study evaluating radiologists' visual assessments reported a sensitivity of 0.92 and a specificity of 0.85 [14] In our study, radiologists’ confidence-based diagnoses achieved a higher AUC of 0.961, with a sensitivity of 0.95 and specificity of 0.86. The high diagnostic performance observed in this study may be attributed to several institution-specific factors. First, our hospital is a high-volume center for lung cancer care that performs many lung cancer surgeries annually. Consequently, pulmonologists, thoracic surgeons, and diagnostic radiologists have extensive experience evaluating pulmonary nodules. Second, the diagnostic process at our institution is not based solely on a single CT scan; rather, it involves a comprehensive assessment that integrates findings from other imaging modalities, patient backgrounds, and clinical course. This multidisciplinary and longitudinal approach likely contributed to the high AUC observed in our study.

Moreover, although previous studies have often employed retrospectively extracted imaging features or semi-automated radiomics approaches [10]. Our analysis was based entirely on narrative radiology reports generated during routine clinical practice. This approach enhances the generalizability and clinical relevance of our findings, as it reflects the way imaging information is actually applied in patient care.

The presence of a GGN was a strong predictor of malignancy in our study, with a sensitivity of 0.984 and a specificity of 1.00. This high diagnostic performance is likely attributable to the well-established association between part-solid GGNs and malignancy, particularly when compared with solid nodules [15]. Although GGNs must be distinguished from inflammatory changes [16], experienced radiologists can often differentiate them with relative ease. Inflammatory lesions typically resolve or evolve over a short follow-up period, whereas malignant GGNs tend to persist or show gradual growth [16]. Thus, the ability to evaluate temporal changes in lesion characteristics is a key factor contributing to the high diagnostic accuracy observed for GGNs.

Diagnostic specificity was also lower (0.779) in patients with coexisting COPD and/or ILD. This finding can be explained by the following two factors: First, parenchymal abnormalities associated with ILD, such as fibrosis and architectural distortion, can obscure nodule margins or mimic malignant features, thereby complicating morphological assessment [17]. Second, both COPD and ILD are established risk factors for lung cancer [18, 19]. Furthermore, lung cancers arising in patients with COPD often lack characteristic imaging features of malignancy, making radiological diagnosis more difficult [20] This complexity may lead radiologists to adopt a more cautious diagnostic approach. In such a high-risk population, radiologists may be less inclined to classify indeterminate nodules as benign, thereby increasing false-positive rates and reducing specificity.

The availability of prior CT scans was associated with improved diagnostic performance, with a sensitivity of 0.953 and a specificity of 0.925. Prior imaging enables the assessment of temporal changes, a fundamental aspect of radiological evaluation. Progressive enlargement of a nodule is a strong indicator of malignancy, whereas a decrease in size or long-term stability supports a benign diagnosis [21–23].

On the other hand, although the AUC for nodules ≤ 1.0 cm in diameter was slightly lower than that for larger nodules, the difference was not statistically significant. This finding can be attributed to two factors. First, the number of small nodules in our dataset may have been insufficient to detect a significant difference in AUC. Second, some small nodules may have GGNs, which are more readily recognized as malignant.

Unexpectedly, PET/CT imaging did not significantly affect diagnostic performance in this study. There are several possible explanations for this finding. The diagnostic utility of PET/CT is limited in small nodules or lesions with low metabolic activity, such as GGNs [24, 25]. In addition, many of the PET/CT scans included in this study were obtained from outside institutions, resulting in variability in imaging quality, acquisition protocols, and reconstruction parameters. This lack of standardization may have reduced the interpretability of the PET/CT findings, thereby limiting diagnostic accuracy.

Although spiculation and pleural indentation were identified as significant predictors of malignancy in the univariate analysis, the overall diagnostic performance, as measured by ROC analysis, was not significantly different based on the presence or absence of these findings. One possible explanation is that the radiologists in the present study did not base their diagnoses on these features in isolation. Instead, they integrated multiple imaging findings including nodule size, shape, margin characteristics, internal attenuation, and temporal changes, to form a comprehensive assessment. As such, even in the absence of spiculation or pleural indentation, other features may compensate for and support an accurate diagnosis. This integrative approach reflects routine radiological practice and may explain why the contribution of any single feature appeared limited in the ROC-based subgroup analysis.

The logistic regression analysis showed that specific imaging features, such as spiculation, as significant predictors of malignancy. However, as demonstrated by the ROC analysis, evaluating these individual findings in isolation does not provide substantial added value compared to the radiologist's overall diagnostic judgment. While the analysis of individual imaging features is valuable for understanding specific predictors of malignancy, these findings do not directly supersede or replace the validity of radiologists’ diagnostic confidence level, which remains the central result of this study.

This study has several important implications for clinical practice. At our institution, radiologists routinely classify confidence levels when determining the benign or malignant nature of pulmonary nodules using a five-point scale, which is included in radiology reports [12]. In this study, the ROM corresponding to each confidence level was calculated. The resulting ROM values showed excellent concordance with the final diagnoses, indicating that the confidence level provided by radiologists is a reliable surrogate for actual diagnostic outcomes. In cytopathology, reporting systems incorporating ROM have been developed to improve patient care by standardizing diagnostic terminology and enhancing communication between pathologists and clinicians [26]. However, ROM estimates vary substantially across institutions and among pathologists. To address this variability, some laboratories have included institution-specific ROM data in their diagnostic reports [26].

Based on this model, we propose that a similar approach may benefit the field of radiology. Specifically, radiology departments could calculate and report institution-specific ROM values for common imaging findings. Incorporating such data into radiology reports may enhance communication with referring clinicians, provide a more transparent understanding of diagnostic certainty, and support shared decision-making in patient management. Such a system would not only improve clinical communication but also contribute to internal quality assurance. By regularly monitoring ROM values and their concordance with final diagnoses, radiology departments can benchmark diagnostic performance and inform educational initiatives for continuous quality improvement.

This study has four limitations. First, it was a retrospective, single-center study conducted at a high-volume tertiary care institution specializing in lung cancer treatment. The cohort was limited to patients referred for diagnostic surgical resection, excluding nodules that were clearly benign on imaging or those that proceeded directly to surgery due to strong clinical suspicion. Consequently, our findings are based on a highly selected preoperative population with a high proportion of malignant cases. This selection bias may have inflated the observed diagnostic performance. Caution is warranted when extrapolating these results to general clinical or screening settings where malignancy prevalence is lower. Further multi-center studies, including low-volume centers, are required to confirm external validity. This will help determine whether our results can be generalized to broader clinical settings beyond high-volume, specialized institutions. Furthermore, there might be another potential selection bias, as patients with chronic renal failure or a history of contrast medium allergy were excluded at the discretion of the attending physician. Second, the confidence levels and descriptive findings were extracted from radiology reports written by experienced radiologists during routine clinical practice. While this reflects routine conditions, it also introduces variability in language and interpretation, as the confidence scale is not based on a standardized reporting template with explicit definitions for each level. Future multicenter studies that incorporate clinical parameters and provide prospective validation are warranted to better assess the generalizability and clinical utility of confidence-based ROM reporting in thoracic imaging. Third, standardized training was not implemented, and inter-rater agreement was not validated in this study. This is because our main objective was to investigate the correspondence between clinical diagnostic confidence and histopathological findings. Forth, the confidence levels described in radiology reports influenced the surgical indication at our institution. Since we compared these reports with the subsequent pathological findings, a degree of circularity may exist in our evaluation.

In conclusion, routine clinical CT-based diagnosis of pulmonary nodules demonstrated high reliability, particularly when interpreted by experienced radiologists within a comprehensive clinical context. Diagnostic accuracy was influenced by objective factors, such as specific imaging features, the availability of prior imaging, and the presence of underlying lung disease. These findings support the value of structured radiology reporting and highlight the potential of confidence-based risk stratification to enhance communication and guide clinical decision-making.

## Data Availability

Data generated or analyzed during the study are available from the corresponding author by request.

## Abbreviations

AUC: Area Under the Curve
COPD: Chronic Obstructive Pulmonary Disease
GGN: Ground-Glass Nodule
ILD: Interstitial Lung Disease
PACS: Picture Archiving and Communication System
ROC: Receiver Operating Characteristic
ROM: Risk of Malignancy
SN: Solid Nodule
